# Changing outcomes following pelvic exenteration for locally advanced and recurrent rectal cancer

**DOI:** 10.1002/bjs5.50153

**Published:** 2019-03-06

**Authors:** PelvEx Collaborative, M. E. Kelly, A. G. J. Aalbers, N. Abdul Aziz, M. Abraham‐Nordling, W. Alberda, A. Antoniou, K. K. Austin, R. Baker, M. Bali, G. Baseckas, B. K. Bednarski, G. L. Beets, P. L. Berg, J. Beynon, S. Biondo, L. Bordeianou, M. Brunner, P. Buchwald, J. W. A. Burger, D. Burling, N. Campain, K. K. L. Chan, G. Chang, M. H. Chew, P. C. Chong, H. K. Christensen, M. Codd, A. J. Colquhoun, A. Corr, M. Coscia, P. E. Coyne, B. Creavin, L. Damjanovic, I. R. Daniels, M. Davies, R. J. Davies, J. H. W. de Wilt, Q. Denost, C. Deutsch, D. Dietz, E. J. Dozois, M. Duff, T. Eglinton, M. Evans, M.D. Evans, N. S. Fearnhead, F. A. Frizelle, E. Garcia‐Granero, J. L. Garcia‐Sabrido, L. Gentilini, M. L. George, R. Glynn, T. Golda, B. Griffiths, J. A.W. Hagemans, D. P. Harji, D. A. Harris, A. A. G. Heriot, W. Hohenberger, T. Holm, J. T. Jenkins, Y. Kanemitsu, S. Kapur, D. S. Keller, S. R. Kelley, H. Kim, C. E. Koh, N. F. M. Kok, R. Kokelaar, C. Kontovounisios, M. Kusters, D. W. Larson, S. Laurberg, W. L. Law, P. Lee, M. L. Lydrup, A. C. Lynch, A. Martling, K. L. Mathis, W. J. H. J. Meijerink, A. M. Mentha, S. Merkel, F. D. McDermott, J. S. McGrath, A. Mihailo, A. Mirnezami, J. R. Morton, T. G. Mullaney, M. B. Nielsen, G. A. P. Nieuwenhuijzen, P. J. Nilsson, P. R. O'Connell, G. Palmer, D. Patsouras, G. Pellino, G. Poggioli, M. Quinn, A. Quyn, R. W. Radwan, S. Rasheed, P. C. Rasmussen, R. Rocha, J. Rothbarth, C. Roxburgh, H. J. T. Rutten, É. Ryan, P. M. Sagar, T. Sammour, A. M. P. Schizas, E. Schwarzkopf, V. Scripcariu, I. Shaikh, D. Shida, A. Simpson, N. J. Smart, J. Smith, M. J. Solomon, M.M. Sørensen, S. R. Steele, D. Steffens, L. Stocchi, N. A. Stylianides, C. Taylor, P. P. Tekkis, S. Tsukamoto, W. H. Turner, J. B. Tuynman, G. H. van Ramshorst, D. van Zoggel, W. Vasquez‐Jimenez, C. Verhoef, M. Verstegen, C. Wakeman, S. Warrier, H. H. Wasmuth, M. R. Weiser, J. M. D. Wheeler, J. Wild, D. C. Winter, J. Yip

## Abstract

**Background:**

Pelvic exenteration for locally advanced rectal cancer (LARC) and locally recurrent rectal cancer (LRRC) is technically challenging but increasingly performed in specialist centres. The aim of this study was to compare outcomes of exenteration over time.

**Methods:**

This was a multicentre retrospective study of patients who underwent exenteration for LARC and LRRC between 2004 and 2015. Surgical outcomes, including rate of bone resection, flap reconstruction, margin status and transfusion rates, were examined. Outcomes between higher‐ and lower‐volume centres were also evaluated.

**Results:**

Some 2472 patients underwent pelvic exenteration for LARC and LRRC across 26 institutions. For LARC, rates of bone resection or flap reconstruction increased from 2004 to 2015, from 3·5 to 12·8 per cent, and from 12·0 to 29·4 per cent respectively. Fewer units of intraoperative blood were transfused over this interval (median 4 to 2 units; *P* = 0·040). Subgroup analysis showed that bone resection and flap reconstruction rates increased in lower‐ and higher‐volume centres. R0 resection rates significantly increased in low‐volume centres but not in high‐volume centres over time (low‐volume: from 62·5 to 80·0 per cent, *P* = 0·001; high‐volume: from 83·5 to 88·4 per cent, *P* = 0·660). For LRRC, no significant trends over time were observed for bone resection or flap reconstruction rates. The median number of units of intraoperative blood transfused decreased from 5 to 2·5 units (*P* < 0·001). R0 resection rates did not increase in either low‐volume (from 51·7 to 60·4 per cent; *P* = 0·610) or higher‐volume (from 48·6 to 65·5 per cent; *P* = 0·100) centres. No significant differences in length of hospital stay, 30‐day complication, reintervention or mortality rates were observed over time.

**Conclusion:**

Radical resection, bone resection and flap reconstruction rates were performed more frequently over time, while transfusion requirements decreased.

## Introduction

Advanced rectal cancer surgery is technically challenging^1^. As outcomes correlate directly with hospital volume^2^, there is a trend towards centralization of rectal cancer resections^3^, ^4^. There is no consensus, however, on the minimum quota for managing complex advanced rectal cancer. Overall referral patterns have remained largely unchanged^5^, as many patients prefer to be treated in their community, even though outcomes may be better in large‐volume centres^6^.

Several studies^4^, ^7^, ^8^ have reported decreased morbidity and mortality for particular complex surgical procedures when performed by surgeons undertaking higher volumes, based on the simplistic premise that ‘practice makes perfect’^9^. Thus, experienced surgeons are thought to be more proficient in terms of technical skill and management of complications^7^. Surgeons' annual volume, rather than cumulative experience, is often reviewed^10^, ^11^ without taking into account the quality of multidisciplinary care available and the effectiveness of perioperative management.

The PelvEx Collaborative was established to provide large‐volume ‘real world’ data on outcomes after pelvic exenteration in specialist tertiary referral units that provide the majority of exenterations for complex rectal cancer in their respective regions. The primary aim of the present study was to investigate trends in surgical outcomes over time, based on data collated from the PelvEx Collaborative. In addition, outcomes were compared between high‐ and low‐volume centres within the collaborative.

## Methods

This was a multicentre retrospective observational study. Patients who underwent pelvic exenteration for locally advanced rectal cancer (LARC) and locally recurrent rectal cancer (LRRC) from 2004 to mid 2015 were eligible. International institutions participated in the collaborative, logging patients in a consecutive series. Each centre is a tertiary referral unit with specialist expertise. All patients were discussed routinely at a dedicated cancer multidisciplinary team meeting. A lead investigator from each participating centre collected data via an institutional database, and data were subsequently submitted centrally for analysis. Ethical approval was obtained and data were processed anonymously.

Before surgery, the diagnosis of rectal cancer was based on histological assessment and/or radiological imaging. An agreed data set was completed by all participating institutions, and data were audited centrally by two reviewers. Any discrepancies were highlighted and referred back to submitting institution for clarification.

First, patient characteristics (age, sex, BMI), differences in bone resection or flap reconstruction (where applicable), margin status (R classification), length of hospital stay, 30‐day major complication rate (Clavien–Dindo grade III or IV) and mortality rate were assessed for trends over the time interval from 2004 to 2015. Second, margin status and extent of resection/reconstruction in high‐volume centres (more than 20 exenterations per year) were compared with those from low‐volume centres (20 or fewer exenterations annually). Twenty pelvic exenterations was the decided as the cut‐off between high‐ and low‐volume after interrogation of the databases, and there was a clear division in centre volume, with several hospitals routinely performing more than 20 exenterations per year, whereas most others showed significant variation in annual volume.

### Statistical analysis

Data were analysed using Prism® 7 (GraphPad Software, San Diego, California, USA). Descriptive analysis was undertaken to report variable frequencies. Categorical variables were analysed using the χ^2^ test, and continuous variables with the Kruskal–Wallis test. Two‐year periods were compared to assess for significant differences. Reported intergroup comparisons were considered statistically significant at the 5 per cent level (*P* < 0·050).

## Results

Some 2472 patients undergoing pelvic exenteration across 26 institutions were included in this study. Male sex was more common in both LARC and LRRC cohorts (60·4 and 63·0 per cent respectively). Patient demographics and operative outcomes are presented in *Tables* 1 and 2 respectively.

**Table 1:** Patient demographics, operative and surgical outcomes following pelvic exenteration for locally advanced rectal cancer, 2004–2015

	2004–2005 (*n* = 142)	2006–2007 (*n* = 172)	2008–2009 (*n* = 218)	2010–2011 (*n* = 250)	2012–2013 (*n* = 340)	2014 to mid 2015 (*n* = 180)
Age (years)*	61	64	63	65	63	63
Sex ratio (M : F)	81 : 61	96 : 76	130 : 88	155 : 95	207 : 133	118 : 62
BMI (kg/m^2^)*	22·7 (18·4–36·1)	23·7 (17·5–43·0)	24·1 (16·3–44·5)	24·3 (15·3–44·3)	24·9 (15·2–43·0)	23 (17·5–40·1)
Bone resection (%)	5 (3·5)	10 (5·8)	16 (7·3)	20 (8·0)	32 (9·4)	23 (12·8)
Flap used (%)	17 (12·0)	35 (20·3)	39 (17·9)	59 (23·6)	97 (28·5)	53 (29·4)
Duration of surgery (min)*	380 (304–480)	340 (245–459)	350 (270–504)	422 (329–555)	425 (313–568)	506 (408–621)
Blood transfusion (units)*	4 (0–22)	3 (0–23)	2 (0–15)	2 (0–24)	2 (0–31)	2 (0–15)
Margin status						
R0	109 (76·8)	130 (75·6)	186 (85·3)	197 (78·8)	266 (78·2)	150 (83·3)
R1	26 (18·3)	27 (15·7)	27 (12·4)	34 (13·6)	34 (10·0)	24 (13·3)
R2	6 (4·2)	7 (4·1)	3 (1·4)	7 (2·8)	6 (1·8)	1 (0·6)
Missing	1 (0·7)	8 (4·7)	2 (0·9)	12 (4·8)	34 (10·0)	5 (2·8)
Length of hospital stay (days)	17 (9–210)	15 (8–120)	14 (7–332)	16 (8–198)	16 (7–104)	18 (6–222)
30‐day major complication rate (%)	64 (45·1)	68 (39·5)	91 (41·7)	86 (34·4)	106 (31·2)	70 (38·8)
Surgical reintervention rate (%)	11 (7·7)	13 (7·6)	22 (10·1)	24 (9·6)	28 (8·2)	13 (7·2)
Radiological reintervention rate (%)	11 (7·7)	12 (7·0)	14 (6·4)	16 (6·4)	17 (5·0)	9 (5·0)
30‐day mortality rate (%)	2 (1·4)	0 (0)	5 (2·3)	3 (1·2)	7 (2·1)	3 (1·7)

Values in parentheses are percentages unless indicated otherwise;

* values are median (range).

**Table 2:** Patient demographics, operative and surgical outcomes following pelvic exenteration for locally recurrent rectal cancer, 2004–2015

	2004–2005 (*n* = 188)	2006–2007 (*n* = 165)	2008–2009 (*n* = 208)	2010–2011 (*n* = 239)	2012–2013 (*n* = 251)	2014 to mid 2015 (*n* = 119)
Age (years)*	63	62	63	62	64	62
Sex ratio (M : F)	123 : 65	112 : 53	134 : 74	146 : 93	145 : 106	77 : 42
BMI (kg/m^2^)*	24·1 (15·0–36·1)	24·2 (15·1–35·5)	25·3 (17·0–37·1)	25·8 (17·8–35·5)	25·5 (18·0–43·1)	24·6 (18·3–30·5)
Bone resection (%)	29 (15·4)	52 (31·5)	49 (23·6)	44 (18·4)	54 (21·5)	21 (17·6)
Flap used (%)	19 (10·1)	27 (16·4)	50 (24·0)	46 (19·2)	37 (14·7)	29 (24·4)
Duration of surgery (min)*	450 (385–575)	524 (421–670)	520 (372–693)	465 (313–644)	480 (356–615)	435 (345–627)
Blood transfusion (units)*	5 (0–28)	5 (0–33)	4 (0–34)	4 (0–34)	3 (0–22)	2·5 (0–21)
Margin status						
R0	108 (57·4)	90 (54·5)	111 (53·4)	147 (61·5)	140 (55·8)	66 (55·5)
R1	54 (28·7)	67 (40·6)	77 (37·0)	71 (29·7)	70 (27·9)	28 (23·5)
R2	14 (7·4)	8 (4·8)	20 (9·6)	13 (5·4)	16 (6·4)	8 (6·7)
Missing	12 (6·4)	0 (0)	0 (0)	8 (3·3)	25 (10·0)	17 (14·3)
Length of hospital stay (days)	17 (8–73)	20 (7–102)	14 (6–189)	14 (7–153)	14 (6–229)	14 (7–78)
30‐day major complication rate (%)	84 (44·7)	60 (36·4)	60 (28·8)	76 (31·8)	77 (30·7)	25 (21·0)
Surgical reintervention rate (%)	16 (8·5)	13 (7·9)	17 (8·2)	20 (8·4)	15 (6·0)	7 (5·9)
Radiological reintervention rate (%)	13 (6·9)	10 (6·1)	11 (5·3)	18 (7·5)	9 (3·6)	5 (4·2)
30‐day mortality rate (%)	2 (1·1)	2 (1·2)	2 (1·0)	5 (2·1)	6 (2·4)	3 (2·5)

Values in parentheses are percentages unless indicated otherwise;

* values are median (range).

### Locally advanced rectal cancer

Some 1302 patients had pelvic exenteration for LARC. Their median age was 63 (21–90) years and median BMI was 23·9 (14–37·4) kg/m^2^. Rates of bone resection and flap reconstruction increased from 2004 to 2015 (bone resection: from 3·5 to 12·8 per cent, *P* = 0·002; flap reconstruction: from 12·0 to 29·4 per cent, *P* = 0·001). There was also an increase in median length of surgery, from 380 to 506 min over the time interval (*P* < 0·001). Median intraoperative blood transfusion decreased from 4 to 2 units during the time period (*P* = 0·040). There was no difference in margin rates across the whole cohort over the 10‐year study period (*P* = 0·766), or in length of hospital stay, 30‐day complication, reintervention or mortality rates.

**Figure 1:**
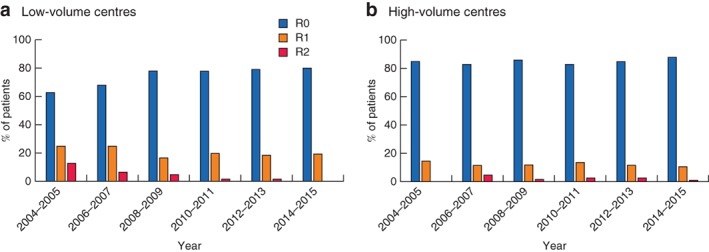
Margin status following pelvic exenteration for locally advanced rectal cancer. **a** Low‐volume centres; **b** high‐volume centres

Subgroup analysis showed that bone resection and flap reconstruction rates increased from 5·2 to 19·5 per cent (*P* = 0·002) and from 13·4 to 34·7 per cent (*P* = 0·001) in high‐volume centres only. Although negative margin (R0) rates improved in both low‐ and high‐volume centres across the time interval, only low‐volume centres had a statistically significant improvement (low‐volume: from 62·5 to 80·0 per cent, *P* = 0·001; high‐volume: from 83·5 to 88·4 per cent, *P* = 0·660) (*Fig*. 1).

### Locally recurrent rectal cancer

Some 1170 patients had pelvic exenteration for LRRC. Their median age was 62 (25–88) years, and median BMI was 24·9 (18·0–34·5) kg/m^2^. The rate of bone resection ranged from 15·4 to 31·5 per cent (*P* = 0·001), and flap reconstruction from 10·1 to 24·4 per cent (*P* = 0·020). Median length of surgery was 472 min, with no change over time. There was a reduction in median intraoperative blood transfusion from 5 to 2·5 units (*P* < 0·001), but no differences in length of hospital stay, 30‐day complication, reintervention or mortality rates.

**Figure 2:**
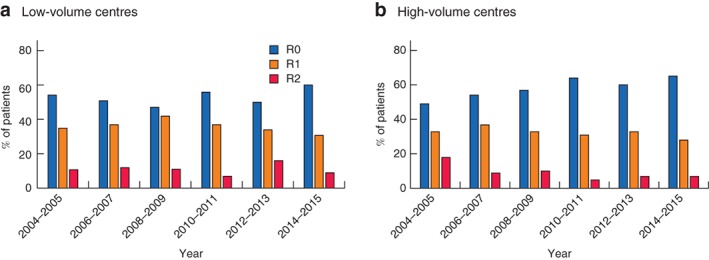
Margin status following pelvic exenteration for locally recurrent rectal cancer. **a** Low‐volume centres; **b** high‐volume centres

Subgroup analysis found no significant differences in bone resection or flap reconstruction rates between high‐ or low‐volume centres over the time. Although not statistically significant, the negative margin rate (R0) increased in both low‐ and high‐volume centres (low‐volume: from 51·7 to 60·4 per cent, *P* = 0·610; high‐volume: from 48·6 to 65·5 per cent, *P* = 0·100) (*Fig*. 2).

## Discussion

With the movement towards centralization of cancer care to optimize patient outcomes, evaluation of the trends in surgical outcomes in centres performing complex rectal cancer surgery is merited. These PelvEx Collaborative data show the trend analysis for outcomes following pelvic exenteration for LARC and LRRC. Improvements in blood transfusion and resection margins status were observed over time. Bone resections to increase radical resections were performed more often, as were flap reconstructions in low‐ and high‐volume centres. These findings reflect improvements in patient selection, better multidisciplinary input, and improvements in overall perioperative care.

The present study did not identify significant changes in patient outcomes in the high‐ *versus* low‐volume centres over time, suggesting that outcomes are not based solely on hospital volume. Liu and colleagues^12^ observed a reduction in 5‐year overall mortality in high‐volume referral centres, but this finding has not been replicated by others^13^, ^14^.

Differences in surgeon experience, diagnostics, multidisciplinary care, (neo)adjuvant therapies and patient factors all have complex effects on surgical and patient outcomes. Case volume *per se* is not a formal indicator of quality, but rather is a structural characteristic^4^, as it appears that organizational expertise, not increased volume, improves patient outcomes. In addition, patients who are willing to travel a long distance to specialist centres tend to be younger, Caucasian, and in higher socioeconomic classes^1^. Elderly patients, or those less able, often remain in local institutions despite the opportunity to attend specialist centres^15^.

A key challenge in assessing volume effect on surgical performance is defining which centre belongs to the high‐ *versus* low‐volume category. In the present study, trends across time were assessed in a group of high‐ and low‐volume centres, rather than comparing specific centres against one another. The authors are aware that not all countries have regionalized exenteration centres. Some countries have lower annual volumes of exenteration surgery owing to population size. Therefore, this study establishes attainable standards for specialist units, and supports the centralization of exenteration surgery to tertiary units that participate in research and international collaboration.

Major limitations of the study include the absence of a definition of individual expertise in pelvic exenteration surgery. Most centres, however, have a number of surgeons with various levels of experience. Management of advanced rectal cancer does not rely on a single individual, but on a surgical unit working together as a team. Owing to the retrospective nature of the study, it was impossible to account for confounding variables such as patient factors, patient selection and diagnostic procedures.

## Collaborators

The following are members of the PelvEx Collaborative: M. E. Kelly, A. G. J. Aalbers, N. Abdul Aziz, M. Abraham‐Nordling, W. Alberda, A. Antoniou, K. K. Austin, R. Baker, M. Bali, G. Baseckas, B. K. Bednarski, G. L. Beets, P. L. Berg, J. Beynon, S. Biondo, L. Bordeianou, M. Brunner, P. Buchwald, J. W. A. Burger, D. Burling, N. Campain, K. K. L. Chan, G. Chang, M. H. Chew, P. C. Chong, H. K. Christensen, M. Codd, A. J. Colquhoun, A. Corr, M. Coscia, P. E. Coyne, B. Creavin, L. Damjanovic, I. R. Daniels, M. Davies, R. J. Davies, J. H. W. de Wilt, Q. Denost, C. Deutsch, D. Dietz, E. J. Dozois, M. Duff, T. Eglinton, M. Evans, M. D. Evans, N. S. Fearnhead, F. A. Frizelle, E. Garcia‐Granero, J. L. Garcia‐Sabrido, L. Gentilini, M. L. George, R. Glynn, T. Golda, B. Griffiths, J. A. W. Hagemans, D. P. Harji, D. A. Harris, A. A. G. Heriot, W. Hohenberger, T. Holm, J. T. Jenkins, Y. Kanemitsu, S. Kapur, D. S. Keller, S. R. Kelley, H. Kim, C. E. Koh, N. F. M. Kok, R. Kokelaar, C. Kontovounisios, M. Kusters, D. W. Larson, S. Laurberg, W. L. Law, P. Lee, M. L. Lydrup, A. C. Lynch, A. Martling, K. L. Mathis, W. J. H. J. Meijerink, A. M. Mentha, S. Merkel, F. D. McDermott, J. S. McGrath, A. Mihailo, A. Mirnezami, J. R. Morton, T. G. Mullaney, M. B. Nielsen, G. A. P. Nieuwenhuijzen, P. J. Nilsson, P. R. O'Connell, G. Palmer, D. Patsouras, G. Pellino, G. Poggioli, M. Quinn, A. Quyn, R. W. Radwan, S. Rasheed, P. C. Rasmussen, R. Rocha, J. Rothbarth, C. Roxburgh, H. J. T. Rutten, É. Ryan, P. M. Sagar, T. Sammour, A. M. P. Schizas, E. Schwarzkopf, V. Scripcariu, I. Shaikh, D. Shida, A. Simpson, N. J. Smart, J. Smith, M. J. Solomon, M. M. Sørensen, S. R. Steele, D. Steffens, L. Stocchi, N. A. Stylianides, C. Taylor, P. P. Tekkis, S. Tsukamoto, W. H. Turner, J. B. Tuynman, G. H. van Ramshorst, D. van Zoggel, W. Vasquez‐Jimenez, C. Verhoef, M. Verstegen, C. Wakeman, S. Warrier, H. H. Wasmuth, M. R. Weiser, J. M. D. Wheeler, J. Wild, D. C. Winter, J. Yip.

## Disclosure

The authors declare no conflict of interest.
